# Long noncoding RNA RP11-838N2.4 enhances the cytotoxic effects of temozolomide by inhibiting the functions of miR-10a in glioblastoma cell lines

**DOI:** 10.18632/oncotarget.9699

**Published:** 2016-05-30

**Authors:** Yanting Liu, Ningbo Xu, Boyang Liu, Yiru Huang, Huijun Zeng, Zhao Yang, Zhenyan He, Hongbo Guo

**Affiliations:** ^1^ The National Key Clinical Specialty, The Engineering Technology Research Center of Education Ministry of China, Guangdong Provincial Key Laboratory on Brain Function Repair and Regeneration, Department of Neurosurgery, Zhujiang Hospital, Southern Medical University, Guangzhou 510282, China

**Keywords:** lncRNA, glioblastoma, chemo-resistance, temozolomide, microarray

## Abstract

Resistance to temolozomide (TMZ), the standard chemotherapy agent for treating glioblastomas (GBM), is a major clinical problem for patients with GBM. Recently, long noncoding RNAs (lncRNAs) have been implicated in chemotherapy resistance in various cancers. In this study, we found that the level of the lncRNA RP11-838N2.4 was lower in TMZ-resistant GBM cells (U87TR, U251TR) compared to the parental, non-resistant GBM cells (U87, U251). In GBM patients, the decreased level of lncRNA RP11-838N2.4 correlated with higher risk of GBM relapse, as well as shorter postoperative survival times. We further found that lncRNA RP11-838N2.4 could enhances the cytotoxic effects of temozolomide to GBM cells both *in vivo* and *in vitro*. Moreover, lncRNA RP11-838N2.4 acts as an endogenous sponge, suppressing the function of miR-10a through conserved sequences and increasing the expression of EphA8 that enhanced the rate of cell apoptosis, thereby intensified sensitivity of GBM cells to TMZ. Additionally, lncRNA RP11-838N2.4 inhibited the activity of transforming growth factor-β (TGF-β) independent of miR-10a. Finally, Characterization of lncRNA RP11-838N2.4 could contribute to strategies for enhancing the efficacy of TMZ.

## INTRODUCTION

Glioblastoma (GBM) is one of the most common malignant tumors in the central nervous system, with a median survival of 15 months after patient diagnosis [1]. Temozolomide (TMZ), an orally administered DNA-alkylating agent, has been the most potent chemotherapy applied in clinic, in addition to surgical excision [2]. However, some patients develop resistance to TMZ, and as such, the overall outcome of GBM patients has not exponentially improved [3]. Predicting the effectiveness of TMZ in patients with GBM and enhancing TMZ sensitivity are therefore of paramount importance. Several studies have indicated that epigenetic modulation of O-6-methylguanine DNA methyltransferase (MGMT) and mismatch repair (MMR) are mechanisms underlying TMZ resistance [2, 4]. Nevertheless, the fact that reduced MGMT levels or stimulated MMR cannot completely prevent TMZ resistance, indicating there are other possible mechanisms that have yet to be elucidated [4, 5].

Long noncoding RNAs (lncRNAs), defined as transcripts of more than 200 nucleotides, influence diverse oncogenic signaling pathways and interact with multiple nucleotides and proteins to regulate cell proliferation, apoptosis, invasion, and vascularization [6]. Increasing research shows that discrepancies lncRNAs levels could contribute to cancer formation and progression, including GBM, carcinoma, leukemia, lung adenocarcinoma, prostate cancer, and colorectal cancer [6, 7]. Moreover, lncRNAs have also been implicated in resistance to various cancer treatments. The lncRNAs ENST00000563280 and NR-036444 induce doxorubicin-resistance in osteosarcoma by increasing the expression of ABCB-1, HIF-1α and FOXC-2 [8, 9]. Additionally, lncRNA UCA1 increases cisplatin resistance in bladder cancer cells by enhancing the activation of Wnt signaling, and lncRNA HOTAIR, which is highly expressed in primary sarcoma, is positively correlated with chemo-resistance [10, 11]. As such, genomic characterization of lncRNA alterations in chemo-resistance may lead to new diagnostic and therapeutic strategies for chemotherapy. Moreover, Chen Y used microarray analysis to summarize the different lncRNAs expression profiles in recurrent and primary gliomas treated with TMZ and found that over 1,100 lncRNAs were distinct expressed between the primary and recurrent glioma [12]. These lncRNAs have potential connections with the progression and pathogenesis of glioma. There are a few lncRNAs that have been confirmed to play a role in TMZ resistance in GBM, though the underlying mechanism is still unclear.

Competing endogenous RNAs (ceRNAs) are important in elucidating how lncRNAs regulate coding genes relative to cancer biology functions [6, 13]. Thousands of lncRNAs act as scaffolds with enormous miRNAs, thus increasing the expression of various genes targeted by miRNAs [14]. For example, lncRNA H19, increase AQP3 expression by sponging miR-874 in the intestinal barrier [15]. Moreover, lncRNA UCA1 contributes to the progression of hepatocellular carcinoma through the inhibition of miR-216b and activation of the FGFR1/ERK signaling pathway [16]. Thus, the different miRNAs decoyed, the different function of lncRNAs exhibited. In previous studies, miR-10a inhibited epithelial-to-mesenchymal transformation (EMT), apoptosis and autophagy, induced chemo-resistance, invasion, proliferation, and tumor growth thought suppressing different protein such as EphA8, BCL2L11, TFAP2C, CDKN1A, p21, and p16 [17, 18]. Notably, miR-10a was up regulated in TMZ-resistant GBM cell and knockdown of miR-10a showed modest cell killing effect in the presence of TMZ [19]. However, whether lncRNAs could reduce TMZ resistance by pairing with miRNAs, such as miR-10a, is still largely unknown.

In our study, we first established TMZ resistance in GBM cell sublines U87TR and U251TR from parent GBM cell lines U87 and U251, respectively [20]. Then, the distinct expression of lncRNAs of U87TR was compared with those of U87 by microarray analysis and we found that lncRNA RP11-838N2.4 was down-regulated. Considering our previous work, we hypothesized that lncRNA RP11-838N2.4 could account for the TMZ resistance by decoying potential miRNAs in an anatomic manner. In this study, we identified the biological function of lncRNA RP11-838N2.4 in TMZ resistance. Besides the occasional finding that lncRNA RP11-838N2.4 inhibited transforming growth factor-β (TGF-β) signaling pathway, it enhanced the rate of cell apoptosis induced by TMZ treatment via miR-10a. Meanwhile, we also demonstrated that lncRNA RP11-838N2.4 level correlated with glioma grading and its lower levels showed higher risk of GBM relapse and shorter postoperative survival time.

## RESULTS

### LncRNA RP11-838N2.4 is a potential TMZ resistance-related lncRNA in GBM

**Figure 1:**
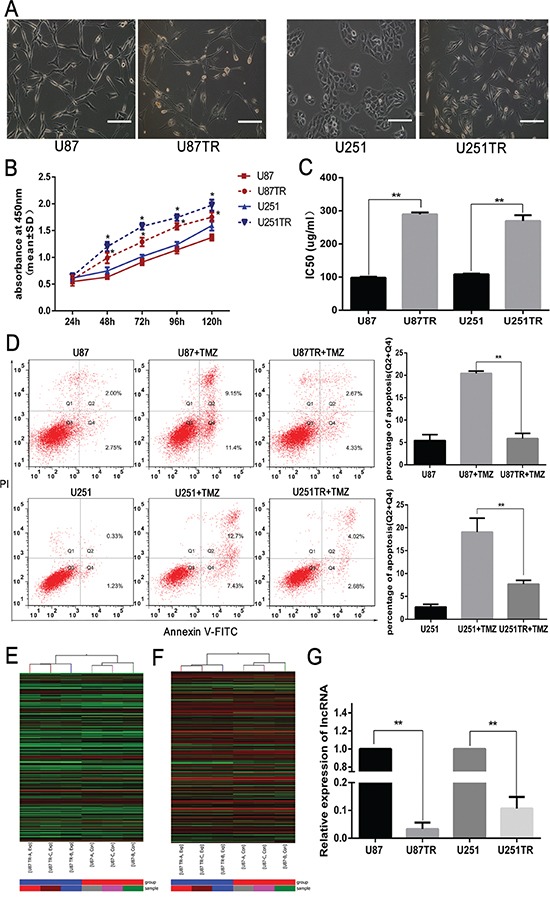
LncRNA RP11-838N2.4 was down regulated in TMZ-resistant GBM cells **A.** Morphological differences between parental GBM cells (U87, U251) and TMZ-resistant GBM cells (U87TR, U251TR). Scale bar, 100 μm. **B.** The cell viability of parental and resistant GBM cells was measured after treatment with TMZ 50μg/ml for 24, 48, 72, 96 and 120 h. **C.** The sensitivities of U87, U87TR, U251 and U251TR cells to TMZ. **D.** Cells were cultured with 0μg/ml or 50μg/ml TMZ for 48h. The apoptosis rate was measured by FACS-based Annexin-V/FITC double staining. Cells positive for Annexin V staining (Q2+Q4) were counted as apoptotic cells. The bar graph shows the percentage of apoptotic cells. **E, F.** Heat map of lncRNAs (E) and mRNAs (F) expression between U87 and U87TR cells. High relative expression is indicated in red and low relative expression in green. The bottom line represents the samples. **G.** qRT-PCR assay of lncRNA RP11-838N2.4 expression in GBM cell lines. TMZ, temozolomide. Data are represented as mean±SD of three independent experiments. *P < 0.05, **P < 0.01.

Using the parental GBM cell lines, U87 and U251, we previously established the TMZ-resistant GBM cell lines, U87TR and U251TR (Figure 1A) [20]. To examine whether U87TR and U251TR were more resistant to TMZ than parent cells, U87 and U87TR, U251 and U251TR were cultured with TMZ 50μg/ml and CCK-8 was performed to assay cell viability. The results showed that U87TR and U251TR were more capable of growth than their parent cells, U87 and U251, when in cultured with TMZ (Figure 1B). Moreover, the half maximal inhibitory concentration (IC50) of TMZ in the U87TR cells (289.53±5.54μg/ml) was higher than U87 cells (98.104±2.63μg/ml), and the IC50 of U251TR cells (269.57±16.98μg/ml) was higher than U251 cells (109.48±1.82μg/ml) (Figure 1C). The rate of cellular apoptosis was also examined by flow cytometry after incubating with TMZ 50μg/ml for 48 hours. The cellular apoptotic rate of the U87TR+TMZ (5.86±1.17%) was lower than U87+TMZ (20.42±0.52%) and that of U251TR+TMZ (7.65%±0.87%) was lower than U251+TMZ (19.05%±3.04%) (Figure 1D). These data indicated that TMZ-resistant U251TR and U87TR cells were less sensitive to TMZ than their parent cell lines.

To screen lncRNAs potentially involved in TMZ resistance, microarray lncRNA analysis was performed with U87TR and U87 cells. 2691 lncRNAs and 4866 mRNAs were significant differentially expressed with statistical significance (fold Change ≥2.0 and *p*-value≤0.05), which shown in Heat map clustering (Figure 1E, 1F). Taking into consideration the fact that the fold changes of lncRNAs were limited by the number of simples and individual characters, the factors about nucleotides number, nearby genes and previous reports of lncRNAs were also needed to be considered for further study. LncRNA RP11-838N2.4 (ENST00000581442), located on chromosome 18: 3466247-3478925, was down-regulated 7.65 fold (*p*-value=0.0002299) in U87TR cells compared to U87 cells. From UCSC Genome Bioinformatics website, we found lncRNA RP11-838N2.4 was one transcript of lncRNA GAPLINC, which mediated cell migration and proliferation by forming a molecular decoy for miR-211 in gastric cancer [21]. Furthermore, TGIF1, an inhibitor of TGF-β signaling, located approximately 400 kb upstream of the lncRNA RP11-838N2.4 gene locus, was also down-regulated, indicating the potential connection between RP11-838N2.4 and TGF-β signaling [22]. Additionally, qRT-PCR showed that the level of lncRNA RP11-838N2.4 was significantly lower in U87TR and U251TR cells compared with parent GBM cells U87 and U251 (Figure 1G). Finally, based on these studies, we hypothesized that the expression of lncRNA RP11-838N2.4 was related to TMZ resistance.

### Down-regulation of lncRNA RP11-838N2.4 correlates with TMZ resistance and poor patient survival in GBM

**Figure 2:**
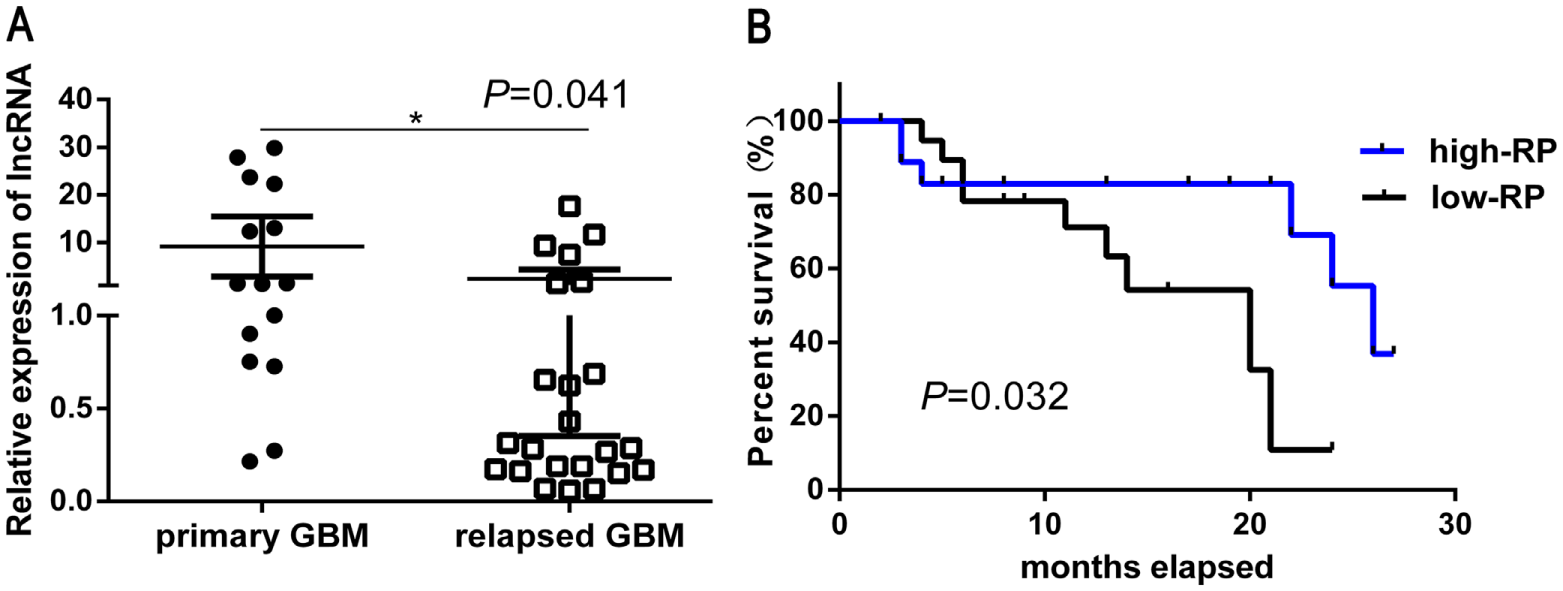
Down regulation of LncRNA RP11-838N2.4 correlates with TMZ resistance and poor patient survival in GBM **A.** Average levels of lncRNA RP11-838N2.4 was detected by qRT-PCR in human primary GBM specimens and relapsed GBM specimens. **B.** Kaplan-Meier overall survival curve according to lncRNA RP11-838N2.4 expression levels in GBM patients (p = 0.032). *P < 0.05, **P < 0.01.

To determine the potential clinical correlation between lncRNA RP11-838N2.4 and TMZ resistance in GBM patients, the expression of lncRNA RP11-838N2.4 was detected by qRT-PCR in patient tissues (15 primary GBM and 23 relapsed GBM). We found that the level of lncRNA RP11-838N2.4 in relapsed GBM patients who were administered TMZ for 6 months was lower than that in primary GBM patients without TMZ treatment (*p*=0.041) (Figure 2A). The relationship between clinical pathology and expression of lncRNA RP11-838N2.4 in patients with glioma were summarized in Table 1. There is no significant association between lncRNA RP11-838N2.4 and the patient's sex or age. However, its expression positively correlated with the tumor grading (WHO I-II vs. WHOIII-IV) (*p*=0.001). Furthermore, Kaplan-Meier analysis depicted that GMB patients with higher level of lncRNA RP11-838N2.4 survived longer (*p*=0.032) than patients with lower level (Figure 2B). Taken together, our results revealed that down-regulation of lncRNA RP11-838N2.4 correlated with TMZ resistance and poor patient survival in GBM.

**Table 1:** Correlation between lncRNA RP11-838N2.4 expression and clinicopathologic characteristics in glioma patients

Characteristics	No	%	Median expression of lncRNA RP11-838N2.4/U6	*P*
Gender				
Male	31	58.5	2.21	0.729
Female	22	41.5	2.11	
Age, year				
<50	16	30.2	2.26	0.078
≥50	50	69.8	2.11	
WHO Grade				
I/II	12	22.7	1.99	0.001^**^
III/IV	41	77.3	2.24	

Abbreviation: WHO=World Health Organization.

** Statistical significance (p<0.01).

### LncRNA RP11-838N2.4 enhance sensitivity of TMZ in U87TR and U251TR GBM cells

**Figure 3:**
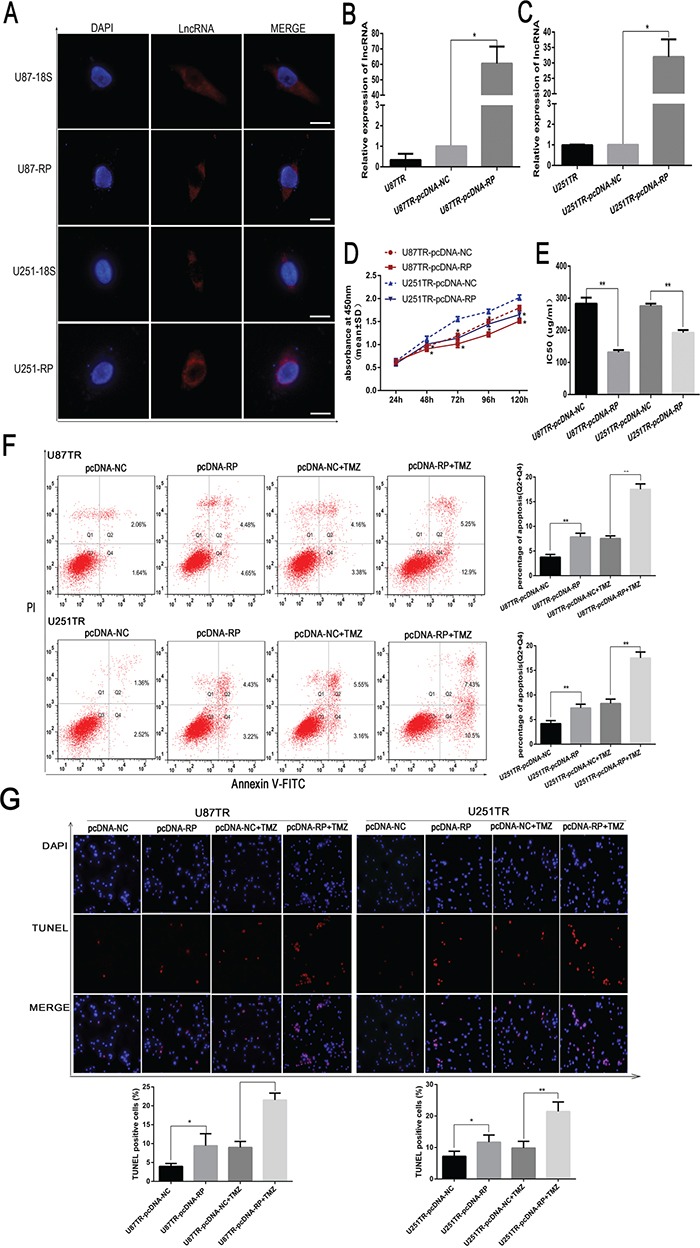
LncRNA RP11-838N2.4 enhances sensitizes of TMZ in U87TR and U251TR **A.** Fluorescent in situ hybridization of the lncRNA RP11-838N2.4 in U87 and U251 cells. DAPI was represented the nuclear and lncRNA 18s probe was used as cytoplasm control. Scale bar: 20μm. **B, C.** the level of lncRNA RP-11-838N2.4 was screened by qRT-PCR in U87TR (B) and U251TR (C) cells that transfected with pcDNA-RP or pcDNA-NC. **D, E.** U87TR and U251TR cells were transfected with pcDNA-RP or pcDNA-NC. The viability was measured by CCK-8 (D) and the sensitivities to TMZ were calculated (E) after GBM cells transfected with indicated constructs. **F, G.** U87TR and U251TR cells were transfected with pcDNA-RP or pcDNA-NC, then cultured with 0μg/ml or 50μg/ml TMZ for 48h. The rate of apoptosis cells was measured by FACS-based Annexin-V/FITC double staining (F) and TUNLE (G). The bar graph shows the percentage of apoptotic cells. (G) Cell nuclear stained with DAPI, and apoptotic cell stained with TUNEL. The TUNEL positive cells were counted in eight randomly selected fields at 200 × magnification. TMZ, temozolomide. Data are represented as mean±SD of three independent experiments.*P < 0.05, **P < 0.01.

To gain insight into the subcellular localization of LncRNA RP11-838N2.4, its probe was designed and RNA FISH was performed in U87 and U251 cells. These data showed that RP11-838N2.4 was mainly distributed in the cytoplasm of the U87 and U251 cell lines (Figure 3A). To ascertain the role of lncRNA RP11-838N2.4 in TMZ sensitivity, we constructed pcDNA vector targeting lncRNA RP11-838N2.4 (pcDNA-RP). The overexpression efficiency was 60.5 fold in U87TR and 31.95 fold in U251TR (Figure 3B, 3C), compared with the non-targeting control (pcDNA-NC). Following transfection with pcDNA-RP in U87TR and U251TR cells, a decrease of cell viability was observed when incubated with TMZ 50μg/ml by CCK-8 assay (Figure 3D). Further, IC50 values of TMZ to pcDNA-RP group were decreased respectively when compared with pcDNA-NC (Figure 3E). Apoptosis was measured by flow cytometry analysis with Annexin V-FITC/PI double staining in GBM cells with TMZ 0μg/ml or 50μg/ml for 48 hours. The results revealed that U87TR and U251TR cells transfected with pcDNA-RP had a moderately higher rate of apoptosis than transfection with pcDNA-NC with TMZ 0μg/ml. This trend was more significant under TMZ 50μg/ml (Figure 3F). The results obtained by TUNEL were consistent with flow cytometry assay (Figure 3G). Collectively, the results showed that up-regulation of lncRNA RP11-838N2.4 enhanced the sensitivity of TMZ and increased apoptosis progression following TMZ treatment in TMZ-resistant GBM cells U87TR and U251TR.

### LncRNA RP11-838N2.4 enhance TMZ-induced cytotoxicity of U87TR *in vivo*

**Figure 4:**
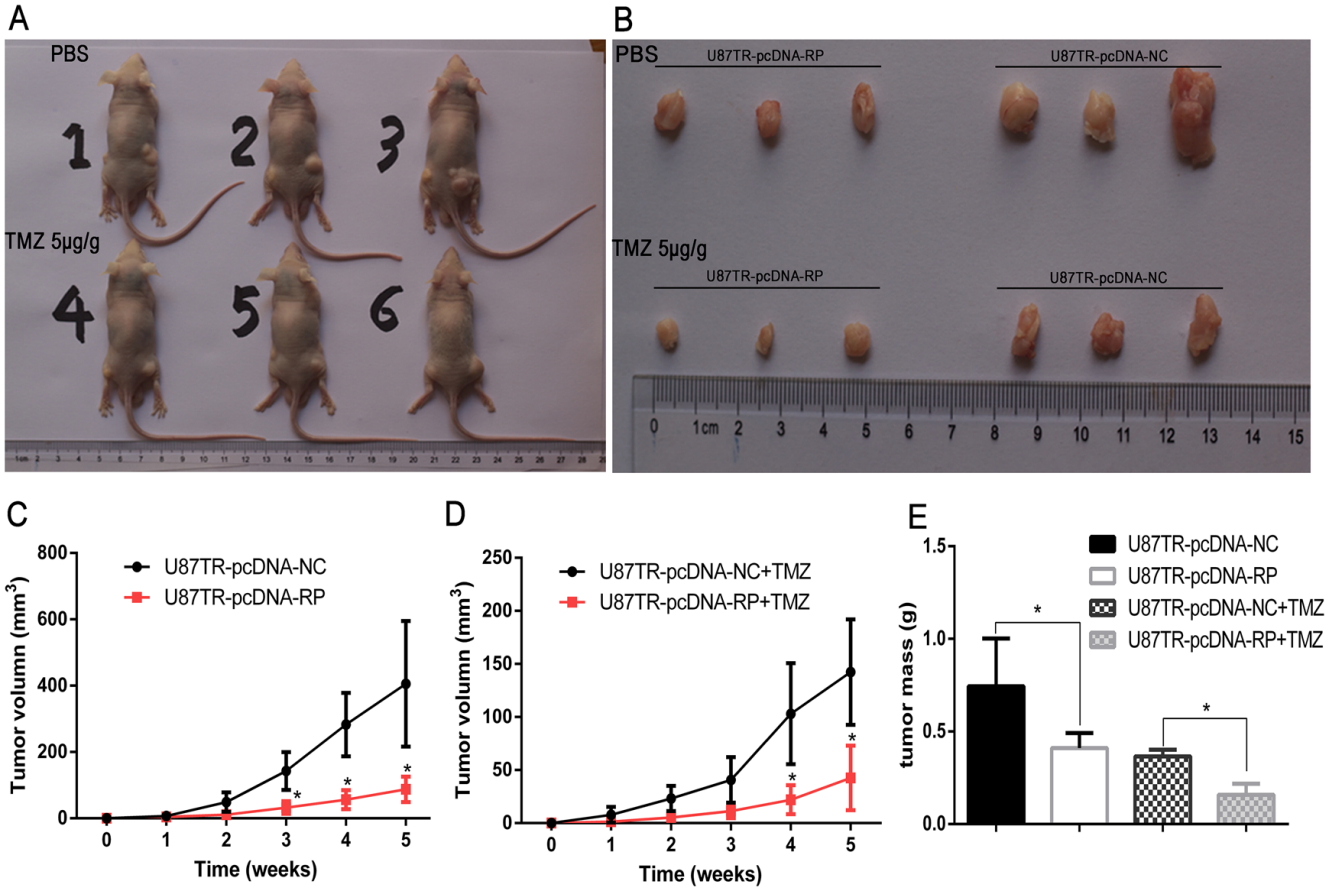
LncRNA RP11-838N2.4 enhances TMZ-induced cytotoxicity of U87TR in vivo **A, B.** Photographs of tumors that developed in xenograft transplanted nude mouse tumor models 5 weeks after injection of U87TR-pcDNA-RP (left side of mouse) or U87TR-pcDNA-NC (right side of mouse). The number of 1-3 nude mouse was treatment with PBS and the number of 4-6 nude mouse was treatment with TMZ 5μg/g. **C, D.** In vivo subcutaneous tumor growth curves were shown for U87TR cells transfected with pcDNA-RP and pcDNA-NC under PBS (C) or TMZ treatment (D). **E.** Weights of xenografts from U87TR cells transfected with pcDNA-RP or pcDNA-NC was measured after 5 weeks. *P < 0.05, **P < 0.01.

To assess the effects of lncRNA RP11-838N2.4 on TMZ-induced cytotoxicity of GBM cells *in vivo*, U87TR cells transfected with pcDNA-NC or pcDNA-RP were synchronously injected into the flanks of nude mice. A xenograft model was applied to measure the size and mass of implanted tumors with treatment of PBS or TMZ 5μg/g via intraperitoneal injection (Figure 4A, 4B). No animals died during the course of the treatment and no other complications, such as skin necrosis due to infection, were detected. The results revealed that, with PBS treatment, U87TR cells transfected with pcDNA-RP showed slower tumor growth compared to U87TR transfected with pcDNA-NC, while this trend was more significant under TMZ treatment (Figure 4C, 4D). After a 5-week inoculation, the average weight of tumors developed from pcDNA-RP-transfected U87TR cells was noticeably smaller than U87TR cells transfected with pcDNA-NC, either with PBS or TMZ treatment (Figure 4E). These results suggest that lncRNA RP11-838N2.4 enhances TMZ-induced cytotoxicity of U87TR cells *in vivo*.

### LncRNA RP11-838N2.4 reduces the expression of miR-10a and relieves its inhibition effect on EphA8

**Figure 5:**
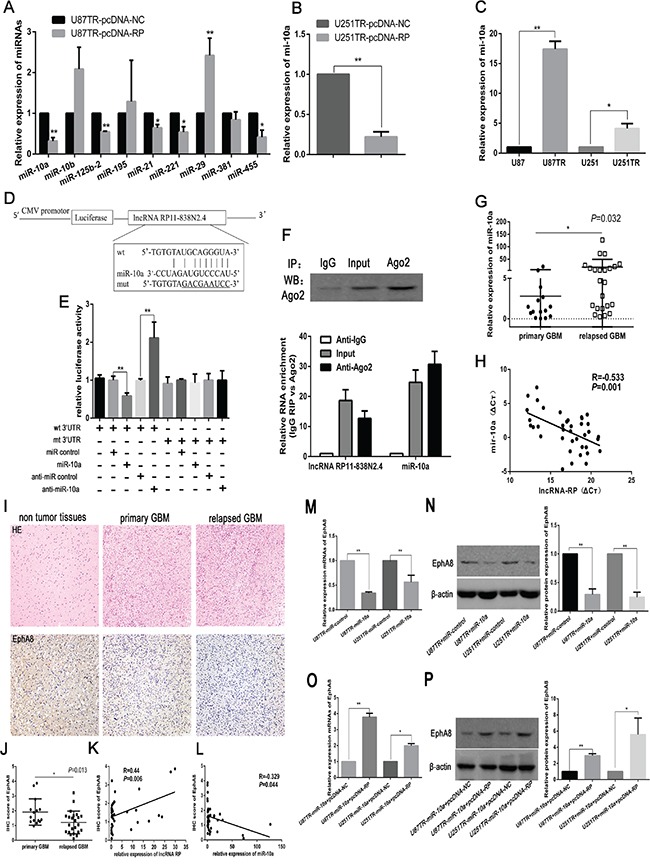
LncRNA RP11-838N2.4 reduces the expression of miR-10a and relieves its inhibition effect on EphA8 **A, B.** Expression of miRNAs was measured by qRT-PCR in U87TR (A) and U251TR (B) cells transfected with pcDNA-RP or pcDNA-NC. **C.** qRT-PCR shows that miR-10a is up regulated in U87TR and U251TR cells. **D.** Sequence alignment of miR-10a with lncRNA RP11-838N2.4. The seed sequence of miR-10a (middle) matches RP11-838N2.4 (top), the mutation of RP11-838N2.4 in luciferase reporter construct (Bottom). **E.** Dual luciferase assay was performed in U87TR cells transfected with luciferase construct alone or co-transfected with miR-10a and anti-miR-10a. Firefly luciferase construct containing mutant target site of the lncRNA RP11-838N2.4 was generated and transfected as indicated. Firefly luciferase activity was normalized to Renillan luciferase activity for each sample. **F.** Cellular lysates from U87TR cells were used for RNA immunoprecipitation (RIP) with Ago2 antibody. Detection of Ago2 performed by IP-western (upper panel), and detection of lncRNA RP11-838N2.4 and miR-10a by qRT-PCR. RNA levels were presented as fold enrichment in Ago2 relative to IgG immunoprecipitates (lower panel). **G.** Average level of miR-10a measured by qRT-PCR in human GBM specimens. **H.** Pearson correlation analysis shows inverse correlation between miR-10a level and lncRNA RP11-838N2.4 level in human GBM specimens. **I.** Expression of EphA8 in human GBM specimen and corresponding non tumorous tissue were assessed by IHC assay. Upper: H&E staining; Lower: immunostaining (×200). Scale bar, 100 μm. **J.** Immunohistochemistry (IHC) scores of EphA8 in primary GBM tissues and relapsed GBM tissues. **K, L.** The correlation analysis between IHC scores of EphA8 and the levels of lncRNA RP11-838N2.4 (K), miR-10a (L) in GBM tissues. **M, O.** The relative mRNAs levels of EphA8 were detected by RT-PCR in U87TR and U251TR cells transfected with indicated constructs and miR-10a. **N, P.** The relative protein levels of EphA8 were detected by western blot analysis in U87TR and U251TR cells transfected with indicated constructs and miR-10a. The relative protein expression levels were obtained from three independent experiments, β-actin was used as a control. Data are represented as mean±SD of three independent experiments. *P < 0.05, **P < 0.01.

To examine whether lncRNA RP11-838N2.4 would regulate miRNAs expression in an anatomic manner, firstly, the candidate miRNAs (miR-10a, miR-10b, miR-125b-2, miR-195, miR-21, miR-221, miR-29, miR-381, and miR-455), which have been validated to play a role in TMZ resistance in glioma, were compared by qRT-PCR in U87TR cells transfected with pcDNA-RP or pcDNA-NC [19, 23–28]. We observed that miR-10a had the greatest down-regulation in U87TR cells transfected with pcDNA-RP (Figure 5A). The level of miR-10a was also down-regulated in U251TR cells transfected with pcDNA-RP (Figure 5B). Additionally, miR-10a was up-regulated in U87TR and U251TR cells, compared with the parent U87 and U251 cells respectively (Figure 5C).

Secondly, the sequences of lncRNA RP11-838N2.4 was compared to miR-10a seed sites by the online software RNA hybird2.0 (Figure 5D). Based on the prediction from this software, reporter constructs containing the wild type sequence of lncRNA RP11-838N2.4 (lncRNA RP11-838N2.4-wt) or its mutant sequence (lncRNA RP11-838N2.4-mut) was co-transfected with miR-10a, miR-control, anti-miR-10a or anti-miR-control into U87TR cells. Transduction of miR-10a caused inhibition of relative luciferase activity of pMIR-lncRNA RP11-838N2.4-wt, but no effect was noticed on pMIR-lncRNA RP11-838N2.4-mut. Meanwhile, miR-10a inhibition by anti-miR-10a increased relative luciferase activity of pMIR-lncRNA RP11-838N2.4-wt compared with anti-miRNA control (Figure 5E). Since miRNAs participated in regulating their targets by facilitating construction of RNA-induced silencing complex (RISC) in AGO2-dependent manner, both lncRNAs and miRNAs might be in the RISC complex. To further define whether lncRNA RP11-838N2.4 decoys miR-10a as molecular sponges, anti-AGO2 RIP was conducted in U87TR cell extracts. Ago2 protein was precipitated by Ago2 antibody in U87TR cellular extract (Figure 5F, upper panel). Furthermore, qRT-PCR showed that levels of lncRNA RP11-838N2.4 and miR-10a in immunoprecipitation were enriched 12.7±2.5 fold and 30.7±4.3 fold in Ago2 pellets compared with IgG immunoprecipitation, respectively (Figure 5F, lower panel). Furthermore, miR-10a was highly expressed in relapsed GBM than primary GBM (Figure 5G) and there was a negative relationship between lncRNA RP11-838N2.4 and miR-10a in GBM tissues, confirmed by qRT-PCR (R=−0.533) (Figure 5H). This result supporting that lncRNA RP11-838N2.4 could interact with miR-10a and reduce its expression.

To explore whether lncRNA RP11-838N2.4 could also relieves miR-10a inhibition effect on its target, the expression of EphA8 was detected (Supplementary Figures S1 and S2) [18]. At first, mRNA and protein level of EphA8 were decreased in U87TR and U251TR cells transfected with miR-10a compared with miR-control (Figure 5M, 5N). Moreover, as predicted, the mRNA and protein level of EphA8 were increased in the U87TR and U251TR cells transfected with miR-10a+pcDNA-RP, compared with the miR-10a+pcDNA-NC (Figure 5O, 5P). At last, the protein level of EphA8 was measured in primary GBM, relapsed GBM tissues, and non-tumor tissues via immunohistochemistry. Compared with primary GBM tissues, the level of EphA8 was lower in relapsed GBM tissues (Figure 5I, 5J). Meanwhile, there was a positive correlation between EphA8 protein level and RP11-838N2.4 expression in GBM tissues (R=0.44, *p*=0.006) (Figure 5K); whereas, there was a negative correlation between EphA8 protein level and miR-10a level in GBM tissues (R=−0.329, *p*=0.044) (Figure 5L). Taken together, these data indicate that lncRNA RP11-838N2.4 could reduce the expression of miR-10a and attenuate its inhibition of downstream targets EphA8.

### LncRNA RP11-838N2.4 reverses the inhibitory effect of miR-10a on TMZ sensitivity of GBM

**Figure 6:**
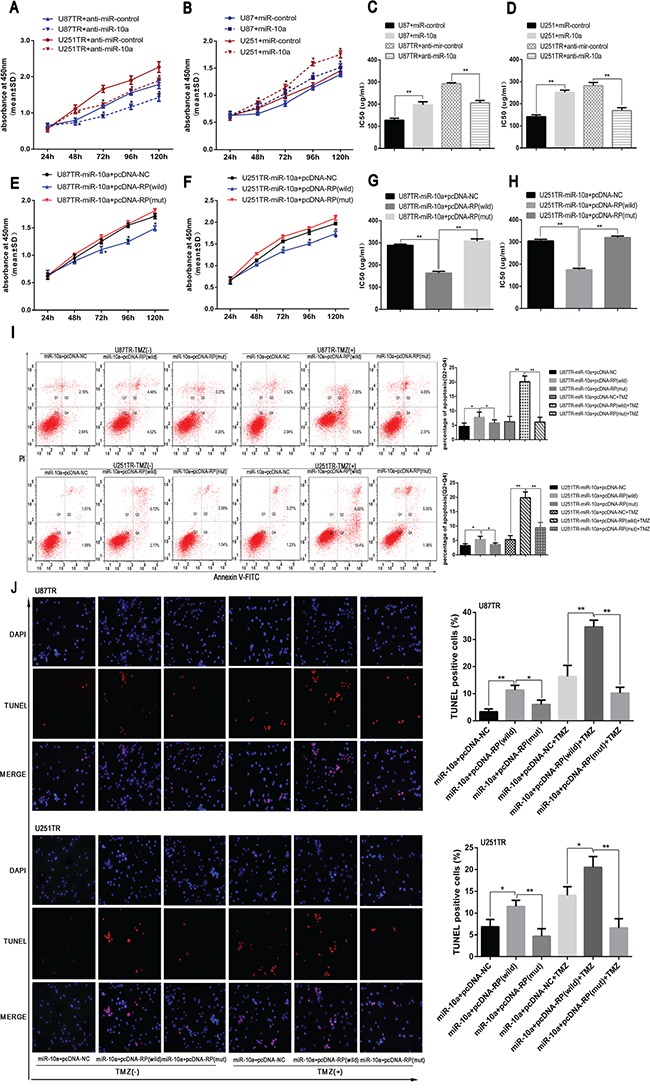
LncRNA RP11-838N2.4 reverses the inhibitory effect of miR-10a on TMZ sensitivity of GBM **A-D.** U87TR and U251TR cells were transfected with anti-miR-10a or anti-miR-control. U87 and U251 cells were transfected with miR-10a or miR-control. The viability was measured by CCK-8 (A, B) and the sensitivities to TMZ were calculated (C, D) after GBM cells transfected with indicated constructs. **E-J.** U87TR and U251TR cells were transfected with miR-10a+pcDNA-NC, miR-10a+pcDNA-RP (wild) or miR-10a+pcDNA-RP (mut). The viability was measured by CCK-8 (E, F) and the sensitivities to TMZ were calculated (G, H) after GBM cells transfected with indicated constructs. Cultured with 0μg/ml or 50μg/ml TMZ for 48h, GBM cells apoptosis rate was measured by FACS-based Annexin-V/FITC double staining (I) and TUNEL (J). The TUNEL positive cells were counted in eight randomly selected fields at 200 × magnification. The bar graph shows the percentage of apoptotic cells. TMZ, temozolomide. *P < 0.05, **P < 0.01.

Since lncRNA RP11-838N2.4 inhibited the expression of miR-10a in GBM, we further investigated whether lncRNA RP11-838N2.4 had the similarity effect on function of miR-10a. CCK-8 showed that cell viability was enhanced in U87 and U251 cells transfected with miR-10a and was decreased in U87TR and U251TR transfected with anti-miR-10a (Figure 6A, 6B). Similarity, IC50 value of U87 and U251 transfected with miR-10a was increased while IC50 value of U87TR and U251TR transfected with anti-miR-10a was decreased (Figure 6C, 6D). These results indicate that miR-10a increased the TMZ resistance.

To confirm that lncRNA RP11-838N2.4 can enhance TMZ sensitivity by interacting with miR-10a in a ceRNA-dependent manner, U87TR and U251TR cells that already over expressed miR-10a were transfected with pcDNA RP-wild, pcDNA RP-mut or pcDNA-NC. Compared with miR-10a+pcDNA-RP-mut and miR-10a+pcDNA-NC, the viability and IC50 value of U87TR and U251TR transfected with miR-10a+pcDNA-RP-wild was decreased (Figure 6E-6H). Additionally, the apoptotic rate of U87TR and U251TR cells transfected with miR-10a+pcDNA-RP-wild was moderately increased compared with the cells transfected with miR-10a+pcDNA-RP-mut or miR-10a+pcDNA-NC under TMZ 0μg/ml (Figure 6I). Interestingly, this trend was significantly amplified under 50 μg/ml TMZ treatment (Figure 6I). TUNEL assay also showed that the apoptotic rate of U87TR and U251TR cells transfected with miR-10a+pcDNA-RP-wild were higher than groups transfected with miR-10a+pcDNA-RP-mut and miR-10a+pcDNA-NC without TMZ treatment, the trend was enlarged with 50μg/ml TMZ treatment (Figure 6J).

All in together, our data shows that lncRNA RP11-838N2.4 could reverse the inhibitory effect of miR-10a on TMZ sensitivity of GBM.

### LncRNA RP11-838N2.4 enhance TMZ sensitivity of GBM in EphA8-dependent manner

**Figure 7:**
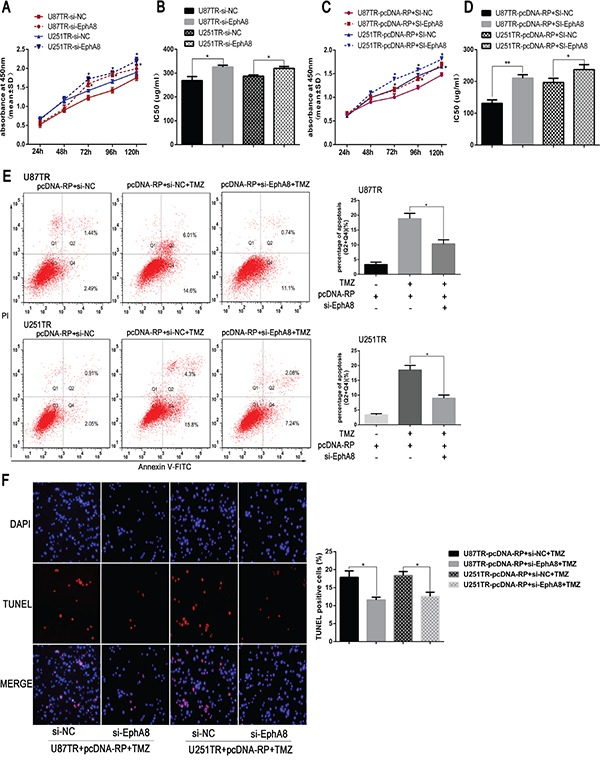
LncRNA RP11-838N2.4 enhances TMZ sensitivity of GBM in EphA8-dependent manner **A, B.** U87TR and U251TR cells were transfected with si-EphA8 or si-NC. The viability was measured by CCK-8 (A) and the sensitivities to TMZ were calculated (B) after U87TR and U251TR cells transfected with indicated constructs. **C-F.** U87TR and U251TR cells were transfected with pcDNA-RP+si-EphA8 or pcDNA-RP+si-NC. The viability was measured by CCK-8 (C) and the sensitivities to TMZ were calculated (D) after U87TR and U251TR cells transfected with indicated constructs. Cultured with 0μg/ml or 50μg/ml TMZ for 48h, U87TR and U251TR cells apoptosis rate were measured by FACS-based Annexin-V/FITC double staining (E) and TUNEL (F). The TUNEL positive cells were counted in eight randomly selected fields at 200 × magnification. The bar graph shows the percentage of apoptotic cells. TMZ, temozolomide. Data are represented as mean±SD of three independent experiments. *P < 0.05, **P < 0.01.

Considering the function of miR-10a depend on its down-stream target and EphA8 played an important role during apoptosis [29], we hypothesize that RP11-838N2.4 reverses the inhibitory effect of miR-10a on TMZ sensitivity of GBM because of attenuations miR-10a inhibition of EphA8. For this purpose, siRNA mediated knockdown of EphA8 was performed in U87TR and U251TR cells. The si-EphA8 and si-NC were transfected and qRT-PCR showed that EphA8 expression level was decreased in si-EphA8-transfected U87TR and U251TR cells compared with cells transfected with si-NC (Supplementary Figure 3). Following transfection with si-EphA8 in U87TR and U251TR cells, an increase of cell viability was observed when incubated with TMZ 50 μg/ml by CCK-8 assay (Figure 7A). Further, IC50 values of TMZ to si-EphA8 groups were increased respectively when compared with si-NC (Figure 7B). This result demonstrated down regulation of EphA8 reduced the sensitivity of TMZ-resistance GBM cell.

To confirmed whether lncRNA RP11-838N2.4 enhanced TMZ sensitivity of GBM in EphA8-dependent manner, U87TR and U251TR cells that already over expressed lncRNA RP11-838N2.4 were transfected with si-EphA8 or si-NC. Compared with pcDNA-RP+si-NC, the cell viability and IC50 value of U87TR and U251TR transfected with pcDNA-RP+si-EphA8 was increased (Figure 7C, 7D). Additionally, the apoptotic rate of U87TR and U251TR cells transfected with pcDNA-RP+si-EphA8, measured by flow cytometry analysis, was moderately decreased compared with the cells transfected with pcDNA-RP+si-NC under TMZ 50μg/ml (Figure 7E). TUNEL assay also showed the similarity result (Figure 7F). All in together, our data indicated that RP11-838N2.4 reversed the inhibitory effect of miR-10a on TMZ sensitivity of GBM, at least in part, because of attenuating miR-10a inhibition of EphA8.

### LncRNA RP11-838N2.4 decreased the activity of TGF-β signaling

**Figure 8:**
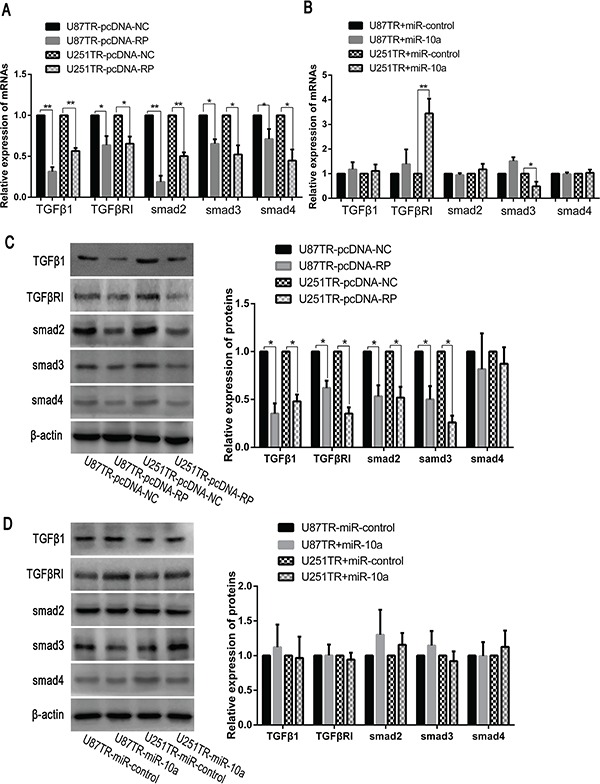
LncRNA RP11-838N2.4 decreased the activity of TGF-β signaling **A, B.** The relative mRNAs level was detected by qRT-PCR in U87TR and U251TR cells transfected with pcDNA-NC or pcDNA-RP (A) and transfected with miR-control or miR-10a (B). **C, D.** The relative proteins level was detected by western blot analysis in U87TR and U251TR cells transfected with pcDNA-NC or pcDNA-RP (C) and transfected with miR-control or miR-10a (D). β-actin was used as a control. Data are represented as mean±SD of three independent experiments. * P<0.05, **P < 0.01.

Previous studies have reported that both miR-10a and TGF-β signaling were important in cancer cell apoptosis and TMZ resistance [19, 30, 31]. To further explore the mechanism linking the lncRNA RP11-838N2.4, miR-10a and TGF-β in TMZ resistance, we examined the mRNA and protein expression levels of TGFβ1, TGFβR1, smad2, smad3 and smad4 in TMZ-resistant cells (U87TR, U251TR) transfected with pcDNA-RP or pcDNA-NC. Compared with U87TR and U251TR cells transfected with pcDNA-NC, the mRNA levels of TGFβ1, TGFβR1, smad2, smad3, and smad4 were reduced in the cell transfected with pcDNA-RP (Figure 8A). The protein level showed the same trend with mRNA expression except smad4 (Figure 8C). Given that up-regulation of lncRNA RP11-838N2.4 could prevent the inhibition of downstream targets of miR-10a, the above mentioned proteins of TGF-β signaling was also detected in TMZ-resistant cells (U87TR, U251TR) transfected with miR-10a or miR-control. However, both the mRNA and protein levels of TGFβ1, TGFβR1, smad2, smad3 and smad4 did not significantly change except the mRNA level of TGFβR1 and smad3 in U251TR transfected with miR-10a (Figure 8B, 8D). Taken together, these data indicate that lncRNA RP11-838N2.4 could inhibit the activity of TGF-β signaling but it is independent on miR-10a.

## DISCUSSION

In this study, we demonstrated that the lncRNA RP11-838N2.4 was down-regulated in the TMZ resistant GBM cells U87TR and U251TR. Moreover, lncRNA RP11-838N2.4 enhanced TMZ sensitivity by inhibiting the expression of miR-10a and increasing EphA 8 expression *in vivo* and *in vitro*. We also found that lncRNA RP11-838N2.4 inhibited TGF-β signaling activity. Importantly, GBM patients with lower lncRNA RP11-838N2.4 expression had an increased risk of recurrence and worse outcomes. This study indicated that lncRNA RP11-838N2.4 had considerable potential in the prognosis and treatment of GBM patients.

Accumulating evidence indicates that lncRNAs contribute to the initiation and development of chemo-resistance. For example, lncRNA HOTAIR increases cisplatin resistance in human lung adenocarcinoma cells [32], lncRNA MALAT-1 increases chemo-resistance to anticancer drugs in pancreatic cancer [33]. Similarity with the profiles analysis of lncRNAs between recurrent gliomas tissue and primary gliomas tissue [12], we noticed thousands of distinct lncRNAs between U87 and U87TR cells. In our study, the level of lncRNA RP11-838N2.4 in TMZ non-resistance cell (U87, U251) was higher than TMZ resistance cell (U87TR, U251TR). Lower level of lncRNA RP11-838N2.4 correlated with higher risk of recurrence and worse outcome of GBM patients. Due lncRNAs display highly heterogeneous effects in different types of cancer, this result indicated the potential prognosis value of lncRNA RP11-838N2.4.

There are multiple functions of lncRNAs involving regulation of gene expression such as epigenomic regulation, chromatin organization, gene transcription and so on [34]. Different with the lncRNAs located in nuclear, which interacting with chromatin modifiers and specific genomic loci, lncRNAs located in cytoplasm can serve as scaffolds to regulate the expression of miRNA target genes through decoy miRNAs [34, 35]. For example, lncRNA CHRF up-regulates Myd88 expression and cardiac hypertrophy by directly binding miR-489 [36]. LncRNA HOTAIR contains a sequence that effectively competes with miR-331-3p, modulating the expression of human epithelial growth factor receptor 2 (HER2) in gastric cancer [37]. In our study, sequence analysis and luciferase reporter assays demonstrated that lncRNA RP11-838N2.4 offered interactions position to miR-10a and had potential act to decoy miR-10a.

Previous studies showed that miR-10a enhanced chemo-resistance in various cancers [27, 38]. For example, the level of miR-10a was increased in DDP-resistant lung cancer cell A549 [38]. Knock down of miR-10a reduced chemo-agent efflux and increased the apoptosis rate of the cell [39]. Furthermore, miR-10a was up-regulated in TMZ-resistant GBM cell [19]. We also found the level of miR-10a was significantly increased in GBM cell U87TR and U251TR cells, concomitant with decreased level of lncRNA RP11-838N2.4. Moreover, we have proven the direct binding ability of the predicted miR-10a binding site on lncRNA RP11-838N2.4 via luciferase reporter assays and RNA-binding protein immune-precipitation assays. Our result also validated that inhibition of miR-10a increasing TMZ sensitivity. These results illustrated that lncRNA RP11-838N2.4 inhibited miR-10a, thereby increasing sensitivity of GBM to TMZ.

In this study, we demonstrated lncRNA RP11-838N2.4 regulated miR-10a distribution on its target and thereby imposed post-transcription regulation. The level of EphA8 was increased when GBM cells transfected with pcDNA-RP. Further interventions revealed that lncRNA RP11-838N2.4 enhanced the cell sensitivity to TMZ treatment through an EphA8-dependent pathway in GBM. Furthermore, this study coincided with another study in which loss of EphA8 resulted in shorter survival times and poor TMZ response in GBM patients [18, 40, 41]. All together, these data demonstrated lncRNA RP11-838N2.4 served as an endogenous sponge, inhibiting both the expression and function of miR-10a. We did not evaluate whether lncRNA RP11-838N2.4 could also inhibit or activate other targets of miR-10a in GBM, such as Survivin, Bcl-2, MDR1, EPHX1, and BRD7 [17]. Further investigation is required.

Considering TGF-β signaling is over-activated during malignant glioma progression, and that it regulates many cellular processes, including apoptosis and cell proliferation [42, 43]. The inhibition of TGF-β signaling might also contribute to the mechanism by which lncRNA RP11-838N2.4 inhibited TMZ resistance. In the present study, lncRNA RP11-838N2.4 reduced mRNA and protein levels of isolated member of TGF-β signaling except smad4 [30]. However, lncRNA RP11-838N2.4 inhibited TGF-β independent of miR-10a. We conclude, therefore, that TGF-β signaling is predominantly regulated by other miRNAs or transcription factors, apart from miR-10a.

Finally, lncRNA RP11-838N2.4 enhanced TMZ sensitivity in GBM by serving as a ceRNA, sequestering with miR-10a on an epigenetic level. This interaction could be considered a potential target for the diagnosis of glioblastoma and outcome assays of TMZ-based therapy. Moreover, the different miRNAs control multiple target genes and lncRNA RP11-838N2.4 may potentially regulate a handful of miRNAs. Several cross-talk signaling pathways are also involved in this regulatory network in GBM. Further elucidating the function of lncRNA RP11-838N2.4 is valuable for identifying and establishing its functional roles in TMZ resistance.

## MATERIALS AND METHODS

### Cell culture

Human GBM cell lines U87 and U251 (gifts from the College of Public Health of Southern Medical University, GuangZhou, China) and its TMZ-resistant variant U87TR and U251TR (established and maintained in our laboratory) were incubated at 37°C in a humidified incubator with an atmosphere of 5% CO_2_ in Dulbecco's modified Eagle's medium (DMEM, Invitrogen), supplemented with 10% fetal bovine serum (Invitrogen), penicillin (200 units/ml) and streptomycin (100 μg/ml). To maintain the TMZ resistance phenotype, U87TR and U251TR were alternately fed with drug-free medium and medium containing 50 μg/ml of TMZ [20].

### Patients and specimens

GBM Patient tissue samples were collected from Zhujiang Hospitals (Southern Medical University, Guangzhou, China) and the stander of Patients enrolled was according the histological diagnosis confirmed. A total of 53 patients were enrolled including thirty-eight GBM cases, three grade III astrocytoma cases, ten grade II astrocytoma cases, two grade I astrocytoma cases, After 6 months temozolomide therapy, twenty-three GBM cases were relapsed. The project protocol was under the approval and guidelines of the Ethics Committee of Zhujiang Hospital and written informed consents were obtained from all of patients enrolled in this study.

### RNA isolation, reverse transcription, and quantitative real-time PCR

Total RNA from tissues or transfected cells was isolated using Trizol reagent (Invitrogen) according to the manufacturer's protocol. The quality and yield of the RNA was examined by the absorbance at 260 and 280 nm. Only samples with an A260:A280 ratio between 1.8 and 2.1 were considered for further analysis. To synthesize cDNA, total RNA was reversely transcribed using prime Script RT reagent Kit (Takara, Japan), miRNAs qPCR Quantitation Kit (Genepharma, Shanghai, China), according to the manufacturer's instructions. The primers for miRNAs (miR-10a, miR-10b, miR-125b-2, miR-195, miR-21, miR-221, miR-29, miR-381, and miR-455) were purchased from RiboBio (Guangzhou, China). Quantitative real-time PCR was carried out in ABI7500 sequence detection system (Applied Biosystems, Foster City, CA, USA) using SYBR Green, according to the manufacturer's protocol. Glyceraldehyde-3-phosphate dehydrogenase (GAPDH) or U6 snRNA was used as an endogenous control. All samples were normalized to internal controls and fold changes were calculated through relative quantification (2^−ΔΔCt^). The primers sequences are shown in Supplementary Table 1.

### Western blot analysis

Total proteins were extracted from cells using radioimmunoprecipitation assay (RIPA) lysis buffer (Sigma-Aldrich) and quantified by Bicin Choninic Acid (BCA) protein assay kit (Thermo). The Western blot was performed according to standard procedures. The following antibodies were used: EphA8 (Abcam, USA), TGFβ1, TGFβR1, smad2, smad3 and smad4 (Cell Signaling Technology, Beverly, MA, USA); β-actin (Proteintech, USA) was used as loading control. The blots were incubated with goat anti-rabbit or anti-mouse secondary antibodies (Bioss, Beijing, China). The proteins were visualized using a chemiluminescence method (ECL Plus Western Blotting Detection System; Amersham Biosciences, Foster City, CA, USA). The bands were quantified by ImageJ software.

### Luciferase reporter assay

We cloned the miR-10a response element (wide type or mutated), contained in the 3′-untranslated regions (3′-UTR) of lncRNA RP11-838N2.4, into target sequence of psiCheck2 plasmid, which is downstream of the luciferase reporter gene. Using a luciferase assay kit (Promega, Madison, WI, USA), luciferase activity was measured and target effect was expressed as relative luciferase activity of the reporter vector with target sequence. After incubating for 48 hours, the cells were lysed in 1x Passive lysis and assayed with the Dual-Luciferase Reporter Assay System (Promega) to measure the Renilla luciferase activity, with firefly luciferase serving as a transfection control.

### TMZ chemo-sensitivity test and IC50 definition

The GBM cells were plated in 96-well plates at 1×10^4^ cells per well after transient transfection or adherence of stable transfected cells. After 24h, the cells were treated with temozolomide (TMZ) (Sigma Chemical Co, St. Louis, MO) concentrations ranging from 40μg/ml to 640μg/ml for 48h. The range of drug concentrations were based on earlier studies and aimed at obtaining IC50 values both for highly sensitive and resistant cases. After incubating with 10 μl of CCK-8 reagent (Dojindo, Molecular Technologies, Dojindo, Japan) for 4h, the absorbance of cells at 450 nm was determined. The spectrophotometric absorbance was then measured using Ultra Multifunctional Microplate Reader (Tecan) at 450 nm. The assay was performed in six replicate wells for each sample and three parallel experiments were conducted simultaneously. IC50 was used to evaluate the resistance of TMZ in the GBM cells.

### Flow cytometric analysis of apoptosis

Cell apoptosis after treatment was evaluated using the Annexin-V/Propidium Iodide Detection Kit (Key GEN, China) according to the manufacturer's instructions. Cells were then analyzed by FACS cytometry (BD Biosciences Inc.)

### Cell transfection

Cells were transiently transfected with 100 nmol/l of miR-10a mimics (miR-203), antagomirs (anti-miR-10a) and miRNA negative control (Genepharma, Shanghai, China) by using Lipofectamine 3000 and OPTI-MEM I (Invitrogen). All of the RNA oligoribonucleotides were purchased from Genepharma (GenePharma, Shanghai, China). Positive transfectants were selected in 500μg/ml Geneticin (G418, Invitrogen). Individual colonies were harvested 24h later for the evaluation of gene expression or functional assays. The full-length of lncRNA RP11-838N2.4, si-EphA8, si-NC was synthesized by RiboBio (Guangzhou, China) and subcloned into the pcDNA3.1 (+) vector (Invitrogen), according to the manufacturer's instructions.

### Tumor growth delay

All investigations were approved by the Animal Experimental Committee of Southern Medical University. Male BALB/C nude mice were purchased from Laboratory Animal Center of Southern Medical University, bred and maintained in a specific pathogen-free facility. For xenograft models: 1×10^6^ U87TR cells, transfected with pcDNA-RP or pc-DNA-NC, were collected and independently injected subcutaneously into the left back and right back of 6 nude mice, respectively. Once tumors were palpable, mice were stratified into two treatment groups. Tumors from one group of mice were injected with TMZ (5μg/g), while the other group was injected with PBS as a vehicle control by intraperitoneal injection every 48h. Mice were anesthetized with 2.5% isoflorane before tumor implantation. Tumor size was monitored routinely for 5 weeks. Mean percent of body weight (±SEM) and tumor size for each group were measured every 7 days.

### Terminal deoxynucleotidyl transferase biotin-dUTP nick end labeling (TUNEL)

Cells were seeded in 6-well plates and treated with TMZ or PBS for 48 hours. Then, apoptosis was determined by a terminal deoxynucleotidyl transferase biotin-dUTP nick end labeling (TUNEL) (Roche, Mannheim, Germany) assay, according manufacturer's protocol. Cells were observed under a fluorescence microscope (Olympus, Japan). The cells with red nuclei staining were defined as apoptotic cells while the cells with blue staining were defined as nuclear. The apoptotic cells were assessed in eight randomly selected fields viewed at 200× magnification.

### Immunohistochemical analysis

Immunohistochemistry for EphA8 was performed on paraffin-embedded GMB or non-tumor samples. Rabbit monoclonal anti-EphA8 antibodies (1:300 dilutions, Abcam) were used as the primary antibodies at 4°C overnight. Then Specimens were incubated with biotinylated secondary antibody (1:500 dilutions, Santa Cruz Biotechnology, USA) 30 min at room temperature. Then the protein was visualized with diaminobenzidine (DAB) and counter staining was conducted with hematoxylin. Staining intensity was scored manually by two independent pathologists as strong staining=3, moderate staining=2, weak staining=1, no staining=0. PBS was used for substituted of EphA8 antibody as negative control. The final immunohistochemistry (IHC) score of specimens was calculated by multiplying the percentage of positive cells with the intensity score.

### RNA immunoprecipitation (RIP) assay

Magna RIP RNA-Binding Protein Immunoprecipitation Kit (Millipore, Billerica, MA, USA) was utilized for RNA immunoprecipitation according the protocol with minor modulation. Mouse IgG (Millipore) was used as negative control, the immune-precipitated RNA was isolated and detected by qRT-PCR. The protein was analyzed by western blotting.

### RNA florescent in situ hybridization (RNA-FISH)

LncRNA RP11-838N2.4 subcellular localizations in GBM cell was analyzed by use of a FISH kit (Roche Applied Science, Germany). The probe of lncRNA RP11-838N2.4 and lncRNA 18S were synthesized by RiboBio (Guangzhou, China). GBM cell were incubated with 4% paraformaldehyde 10 min at room temperature, then with a digoxin-labeled lncRNA RP11-838N2.4 probe overnight. The lncRNA 18S probe was used as cytoplasm control. Cell nuclei were stained with 4 ′,6-diamidino-2-phenylindole (DAPI). Cells were observed under a fluorescence microscope (Olympus, Japan).

### Statistical analysis

SPSS 20.0 software (SPSS Inc., Chicago, IL, USA) and Graph Pad Prism software (Graph Pad Software, Inc., La Jolla, CA, USA) were used to analyze all data for statistical significance. The Chi-Square test was applied to the examination of the relationship between lncRNA expression levels and clinic pathological characteristics. Two-tailed Student's t-test was used for comparisons of two independent groups. One-way ANOVA was used to determine the differences between groups for all *in vitro* analyses. Statistical significance was set at *P < 0.05, **P < 0.01. P < 0.05 was considered to indicate statistical significance.

## SUPPLEMENTARY FIGURES AND TABLE


