# Supplementary material for: Long noncoding RNA RP11-838N2.4 enhances the cytotoxic effects of temozolomide by inhibiting the functions of miR-10a in glioblastoma cell lines
Source: Oncotarget. 2016 May 30;7(28):43835–51. doi: 10.18632/oncotarget.9699 (PMC5190063; doi:10.18632/oncotarget.9699)
Supplement: Supplementary file 1 [file oncotarget-07-43835-s001.pdf]

Long noncoding RNA RP11-838N2.4 enhances the cytotoxic effects of temozolomide by inhibiting the functions of miR-10a in glioblastoma cell lines

SUPPLEMENTARY FIGURES AND TABLE

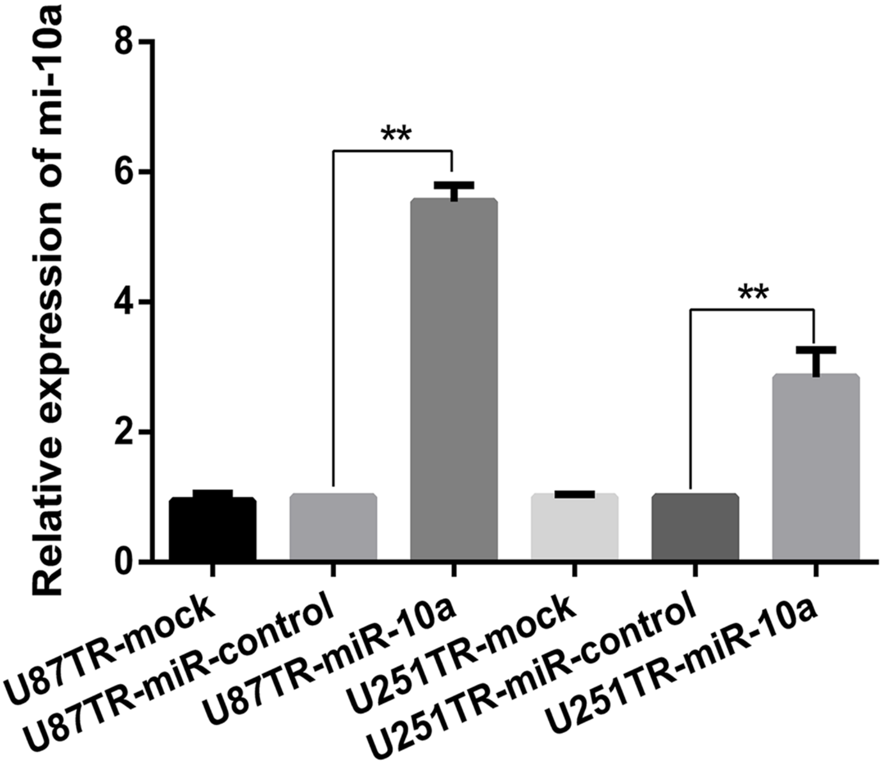

Supplementary Figure S1: The expression of miR-10a was detected by qRT-PCR in U87TR and U251TR cells transfected with miR-control or miR-10a. **P < 0.01.

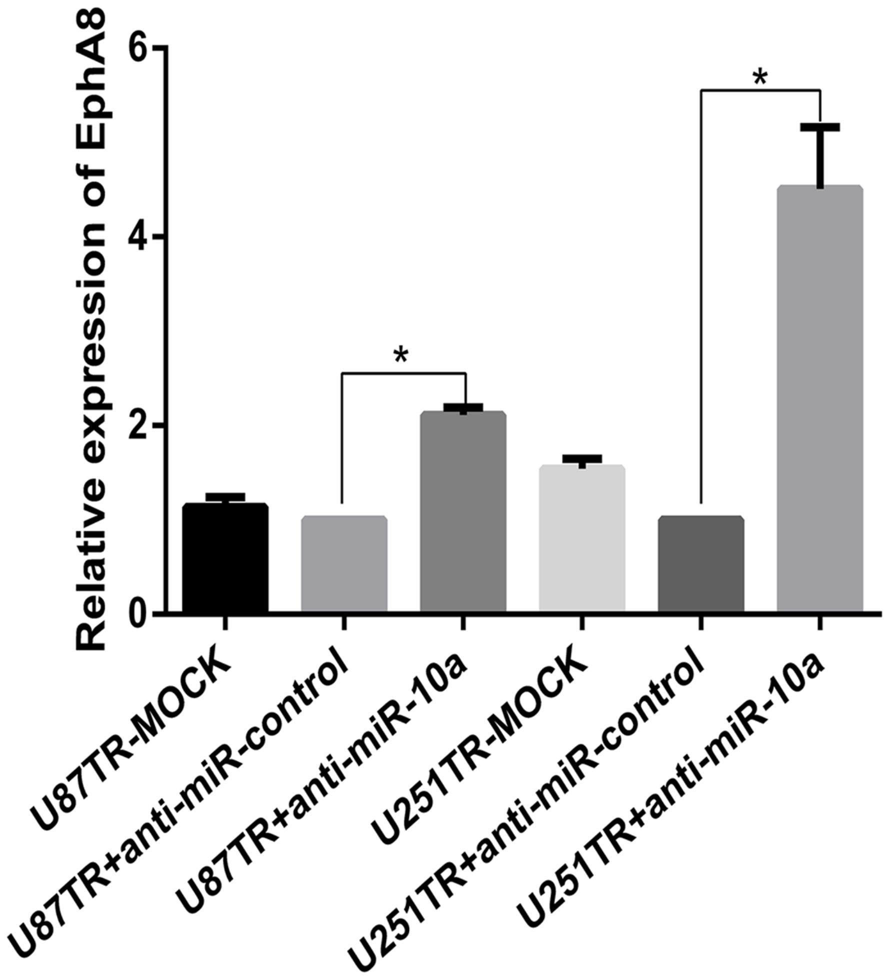

Supplementary Figure S2: The mRNA level of EphA8 was detected by qRT-PCR in U87TR and U251TR cells transfected with anti-miR-control or anti-miR-10a. *P < 0.05.

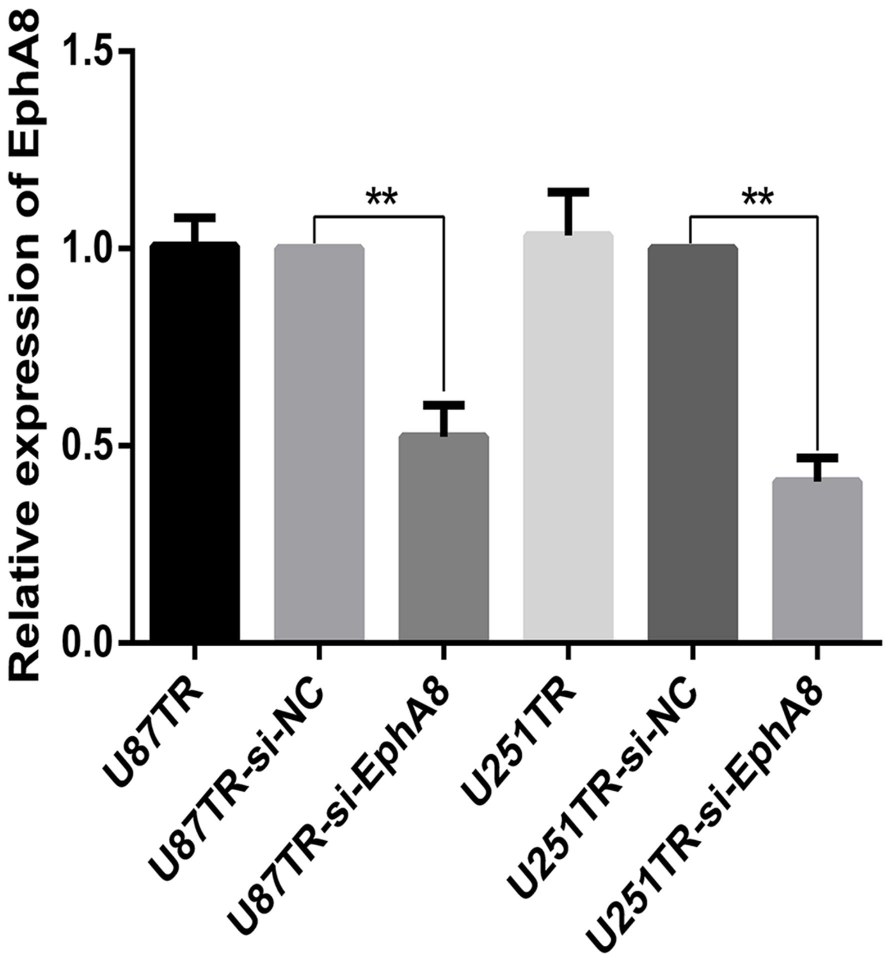

Supplementary Figure S3: The mRNA level of EphA8 was detected by qRT-PCR in U87TR and U251TR cells transfected with si-NC or si-EphA8. **P < 0.01.

Supplementary Table S1: The primers used in qPCR analysis

lncRNA/mRNA seqname	Forward primer	Reverse primer
lncRNA RP11-838N2.4	GTTTCCTGGAAGGGCATTTT	TCCAGCTTCTCCTTTTGCA
U6	CTCGCTTCGGCAGCACA	AACGCTTCACGAATTTGCGT
EphA8	GCGCGTCTATGCTGAGATCAA	CGGTCCGACTCCAGGTAGT
TGFβ1	TACAGCACGGTATGCAAGCC	GCAACCGATCTAGCTCACAGAG
TGFβR1	ACGGCGTTACAGTGTTTCTG	GCACATACAAACGGCCTATCTC
smad2	CCGACACACCGAGATCCTAAC	GAGGTGGCGTTTCTGGAATATAA
smad3	TGGACGCAGGTTCTCCAAAC	CCGGCTCGCAGTAGGTAAC
smad4	CTCATGTGATCTATGCCCGTC	AGGTGATACAACTCGTTCGTAGT
GAPDH	TGTGGGCATCAATGGATTTGG	ACACCATGTATTCCGGGTCAAT
